# Poly(Ionic) Liquid‐Enhanced Ion Dynamics in Cellulose‐Derived Gel Polymer Electrolytes

**DOI:** 10.1002/cssc.202401710

**Published:** 2024-11-26

**Authors:** Tiago G. Paiva, Maykel Klem, Sara L. Silvestre, João Coelho, Neri Alves, Elvira Fortunato, Eurico J. Cabrita, Marta C. Corvo

**Affiliations:** ^1^ I3N Cenimat Department of Materials Science (DCM) NOVA School of Science and Technology NOVA University of Lisbon Caparica 2829-516 Portugal; ^2^ Centro de Química Estrutural Institute of Molecular Sciences and Departamento de Engenharia Química Instituto Superior Técnico, Universidade de Lisboa, Avenida Rovisco Pais Lisboa 1049-001 Portugal; ^3^ School of Technology and Sciences São Paulo State University (UNESP) Presidente Prudente SP 19060-900 Brazil; ^4^ Dpto. Física de la Materia Condensada Instituto de Ciencia de Materiales de Sevilla (Universidad de Sevilla-CSIC Avda. Americo Vespucio 49 41092 Sevilla Spain; ^5^ UCIBIO Department of Chemistry NOVA School of Science and Technology NOVA University of Lisbon Caparica 2829-516 Portugal

**Keywords:** Cellulose, Poly(Ionic liquids), Supercapacitors, Energy storage, Nuclear magnetic resonance

## Abstract

Gel polymer electrolytes (GPEs) are regarded as a promising alternative to conventional electrolytes, combining the advantages of solid and liquid electrolytes. Leveraging the abundance and eco‐friendliness of cellulose‐based materials, GPEs were produced using methyl cellulose and incorporating various doping agents, either an ionic liquid (1‐Butyl‐1‐methylpyrrolidinium bis(trifluoromethylsulfonyl)imide [Pyr14][TFSI]), its polymeric ionic liquid analogue (Poly(diallyldimethylammonium bis(trifluoromethylsulfonyl)imide) [PDADMA][TFSI]), or an anionically charged backbone polymeric ionic liquid (lithium poly[(4‐styrenesulfonyl)(trifluoromethyl(S‐trifluoromethylsulfonylimino) sulfonyl) imide] LiP[STFSI]). The ion dynamics and molecular interactions within the GPEs were thoroughly analyzed using Attenuated Total Reflectance Fourier‐Transform Infrared Spectroscopy (ATR‐FTIR), Heteronuclear Overhauser Enhancement Spectroscopy (HOESY), and Pulsed‐Field Gradient Nuclear Magnetic Resonance Diffusion (PFG‐NMR). Li^+^ transference numbers (t_Li_+) were successfully calculated. Our study found that by combining slow‐diffusing polymeric ionic liquids (PILs) with fast‐diffusing lithium salt, we were able to achieve transference numbers comparable to those of liquid electrolytes, especially with the anionic PIL, LiP[STFSI]. This research highlights the influence of the polymer′s nature on lithium‐ion transport within GPEs. Additionally, micro supercapacitor (MSC) devices assembled with these GPEs exhibited capacitive behavior. These findings suggest that further optimization of GPE composition could significantly improve their performance, thereby positioning them for application in sustainable and efficient energy storage systems.

## Graphical abstract

Advancing Energy Storage: Gel Polymer Electrolytes with Promising Potential Gel polymer electrolytes (GPEs) incorporating methyl cellulose and a range of ionic/polymeric achieved Li^+^ transference numbers comparable to traditional liquid electrolytes. These GPEs exhibited exceptional potential for employment in microsupercapacitors (MSCs), pointing towards an exciting frontier in sustainable and efficient energy storage systems.

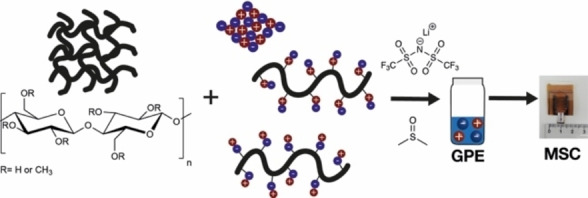

## Introduction

Supercapacitors (SC) are a class of electrolytic energy storage devices (EESD) that comprise Li‐ion batteries and fuel cells. SC are an emergent technology that provides fast response times and high‐power output being suitable for applications where short bursts of energy are required[^1^, ^2^] Regular capacitors are composed of two electrodes separated by a dielectric layer; upon the presence of an electrical field, the device stores energy by accumulating charges at the interface. SC differ from this technology by replacing the dielectric with an electrolyte where electrical double layers can be formed.^[3]^ Gel or solid‐state polymer‐based electrolytes are generally preferred for wearable and flexible devices as they prevent harmful electrolyte leakage. In this context, cellulose, an abundant and biodegradable polymer with mechanical characteristics, is suitable for developing new sustainable materials for several applications, including energy storage devices. However, its low solubility in common organic solvents remains a challenge. A common strategy to address this issue is its derivatization into more processable materials.^[4]^ There are several cellulose derivatives readily available commercially; one of those is methyl cellulose (MC) a cellulose ether that is soluble in water and polar aprotic organic solvents such as dimethyl sulfoxide (DMSO) and dimethylformamide (DMF).[^5^, ^6^] The solution impregnation method is extensively employed in the fabrication of gel polymer electrolyte (GPE) membranes, MC can produce thermoreversible gels in these solvents and is used in this work to produce gel polymer electrolytes (GPE) for supercapacitor applications.

The process of MC gelation has been subject to research, and several mechanisms have been proposed. These mechanisms include physical crosslinking of MC chains,[^7^, ^8^, ^9^] micelle formation,[^10^, ^11^, ^12^] and phase separation‐induced gelation.[^13^, ^14^, ^15^, ^16^, ^17^, ^18^] From transmission electron cryomicroscopy (cryo‐TEM) images,^[19]^ it was possible to prove that, upon heating, MC chains assemble into a fibril network.[^20^, ^21^, ^22^] These results have been confirmed by small‐angle neutron scattering (SANS) and small‐angle X‐ray scattering (SAXS) experiments,[^19^, ^21^] fitting well to a flexible cylinder model with a diameter of 14 to 20 nm. The gelation process in water is believed to undergo an initial stage resulting in a transparent, fragile gel. Subsequently, at elevated temperatures, it forms a turbid, strong gel due to phase separation.[^18^, ^23^] The process undergoes several stages from random MC coils in solution to assembling into a 3D network of either coils and helixes and/or beads and rods. The assembly is a statistical process described mathematically elsewhere.^[24]^ Tanaka *et al*.^[25]^ described the process as follows: first MC chains form helices and then bundle to form the gel while cooling. The bundling of otherwise random coils is due to hydrophobic forces between the O‐CH_3_ groups, which are affected by factors such as the polymer molecular weight, degree of substitution (DS), and the presence of salts or surfactants. Dynamic mechanical analysis (DMA) measurements revealed two different assemblies’ cohesion, with two different entanglement bonding strengths.^[26]^

The gel formation in organic solvents is known to have a different mechanism. First, MC is dissolved during the heating cycle in a solvent such as DMF, dimethylacetamide (DMAc) or DMSO, and then, the gelation occurs during the cooling phase. While the assembly steps for the sol‐gel transition in water are well known, in organic solvents its study is much more recent.[^27^, ^28^, ^29^, ^30^, ^31^] Singh and Kundu studied the formation of MC gels in DMF using rheology,^[29]^ and differential scanning calorimetry (DSC) measurements.^[28]^ At higher temperatures, MC gels in water become cloudy, a phenomenon not observed in organic solvent gels that also do not exhibit phase separation. The polarized optical microscopy (POM) observed turbidity at high temperatures is due to MC producing larger beads.^[28]^ The same authors reported that DMF′s capability to establish H‐bonds with MC′s O‐CH_3_ and OH groups is responsible for the different gelation regimes.^[27]^ DSC measurements show that, while heating, the increased energy in the system leads to an enhancement of MC–DMF interactions, contributing to the overall sol‐gel transition. Whereas the coil‐helix model can describe the MC‐water gels, MC–DMF gels are better explained by a coil‐helix‐bead‐rod model,^[28]^ where the helixes form beads while cooling rather than heating and then further aggregate to form rods after heating.

Ionic liquids (ILs) are a class of materials composed of an organic cation and an organic or inorganic anion. Their unique properties, including low flammability, high ionic conductivity, thermal stability, high heat capacity, negligible volatility, and strong polarizability, make them stand out in the field of energy storage. Additionally, their solubilizing effects and tunability further enhance their appeal. Due to their electrochemical properties, low flammability, and negligible vapor pressure, ILs[^32^, ^33^] and deep eutectics solvents (DES),[^34^, ^35^] which share some of the ILs characteristic, such as high thermal stability and high ionic conductivity are desirable candidates for high‐performance electrolytes. The evolution of poly (ionic liquid)s (PILs), which combine the advantageous properties of ILs with the mechanical strength of polymers, represents a significant advancement in energy storage. This synergy aims to enhance the performance of energy storage devices.

**Figure 1:**
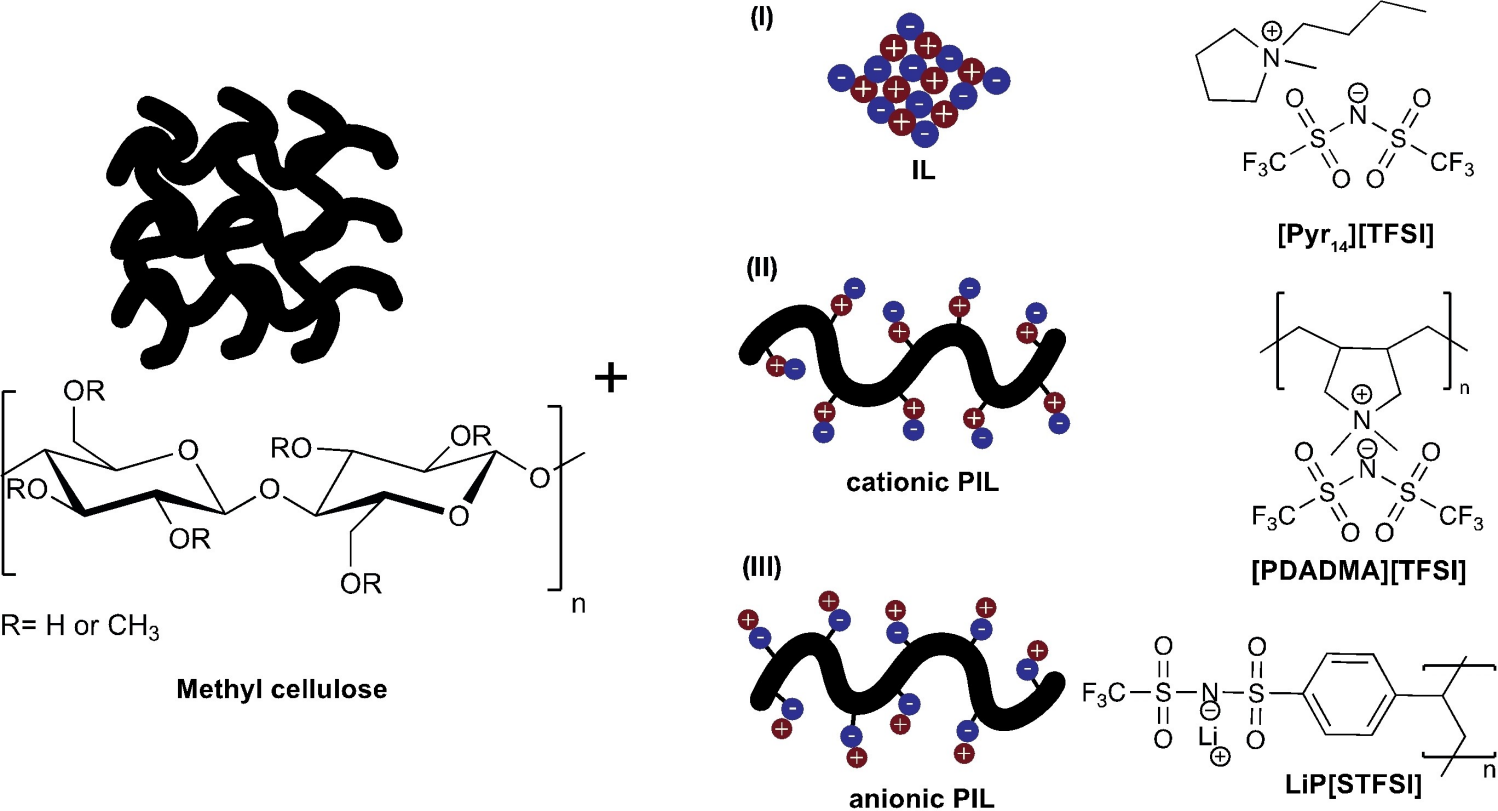
Structure of methyl cellulose and the IL/PILs used in this work GPE formulation.

In the present work, we explored MC′s ability to form thermoreversible gels in organic solvents to prepare GPEs, using novel formulations with different PILs (Figure 1). GPEs containing PILs can offer enhanced ionic conductivity, improved mechanical stability, and greater thermal resistance compared to traditional electrolytes, making them ideal for advanced energy storage applications.^[36]^ Here, the effects of using a PIL against an IL with similar structural features and using a PIL with an anionic backbone were studied. The obtained gels were implemented for SC assembly and testing as proof of concept.

## Experimental Section

MC GPEs were prepared using 1‐Butyl‐1‐methylpyrrolidinium bis(trifluoromethylsulfonyl)imide, ([Pyr14][TFSI]. MC was initially dissolved in DMSO or (DMSO‐*d_6_*) and then the IL was added in a 10/90 wt. ratio. In the sample with lithium bis(trifluoromethylsulfonyl)imide, LiTFSI, the salt was first dissolved in the IL. MC GPEs with PIL samples were prepared by dissolving MC in DMSO‐*d_6_* followed by the addition of Poly(diallyldimethylammonium bis(trifluoromethylsulfonyl)imide, P[DADMA][TFSI], or lithium poly[(4‐styrenesulfonyl)(trifluoromethyl(S‐trifluoromethylsulfonylimino)sulfonyl)imide], LiP[STFSI], and then LiTFSI to afford a lithium concentration on the range of 0.2 to 2.0 M. Sample composition is presented in Table 1. The synthetic methodology for the preparation of [PDADMA][TFSI] and LiP[STFSI] and further experimental details can be found in the supporting info.

**Table 1:** Composition (wt. %) of the studied GPEs.

Sample	MC	DMSO	LiTFSI	IL/PIL
MC_[Pyr 14][TFSI]_Li 0.2 M	2.1	78.4	4.1	19.2
MC_[Pyr_14][TFSI]_Li 1.0 M	1.8	65.0	17.0	16.2
MC_[Pyr_14][TFSI]_Li 1.5 M	1.7	60.6	29.1	14.9
MC_[Pyr_14][TFSI]_Li 2.0 M	1.5	55.1	29.8	13.6
MC_P[DADMA][TFSI]_Li 1.0 M	1.8	64.7	17.5	16.0
MC_P[DADMA][TFSI]_Li 1.5 M	1.6	58.3	24.5	14.6
MC_LiP[STFSI]_Li 1.0 M	1.4	78.0	19.9	0.7
MC_Li 1.0 M	2.1	78.4	19.5	0.0

**Figure 2:**
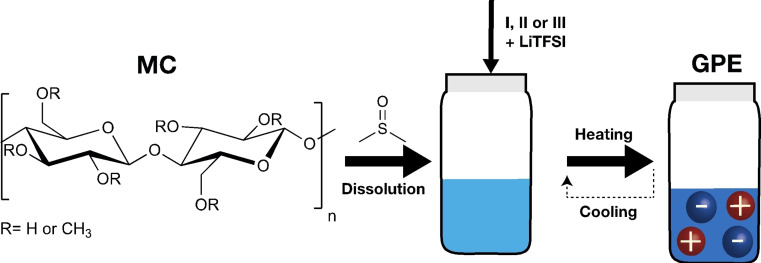
Overview of the methodology to obtain MC GPEs.

The GPEs were obtained through heating/cooling cycles until the gel was formed upon cooling the mixture, as shown in Figure 2.

## Results and Discussion

Several commercially available cellulose derivatives are soluble in common organic solvents. MC generates gels in water and some organic solvents.[^27^, ^37^, ^38^] Lately, our research has focused on exploring the interactions between cellulose and ILs.^[39]^ In this work, we prepared MC gels in DMSO with IL/PILs to investigate the ion dynamics in these systems (Figure 2). After a thorough characterization to better understand the transport properties of these GPEs, we tested them in SC. The choice of ILs and PILs aims to investigate their impact on lithium‐ion mobility. However, it is only possible to analyze this effect by dealing with the interaction between ILs and cellulose,^[36]^ including the impact of salts on gel temperature, specifically the influence of anions on gelation temperature.

Several studies have been conducted to understand how salts affect the decrease or increase in gel temperature. Based on their effects, these studies often refer to the Hoffmeister series and classify salts as kosmotropic (structuring) or chaotropic (destructuring). Anions with dehydrating effects can cause salting‐out of the polymer, lowering the gel temperature, while chaotropes can increase the gelation temperature due to their hydrophilic interactions.^[40]^ However, most of these studies are carried out in water rather than an organic solvent.[^6^, ^18^] Liu *et al*. studied the effect of salt addition to a carboxymethyl cellulose (CMC) aqueous gel in the presence of [C_12_C_1_Im]Br.^[41]^ The long chain length of the IL and its capability to produce micelles greatly influence the solution dynamics. At low NaBr concentration, [C_12_C_1_Im]^+^ binds to the cellulose chains, but with the increase in IL concentration, micelles form and percolate the polymer chains. At a sufficiently high concentration of NaBr, micelle formation occurs without forming IL–CMC complexes.

ILs have been used previously with MC and other cellulosic derivatives to produce energy storage solutions. Chinnam *et al*. used MC_[Pyr14][TFSI] based solid ion‐gel separators, achieving a high Li^+^ transference number of 0.36 and mS level of r.t. conductivity by doping the material with a tetrameric anionic siloxane.^[42]^

In the present work, the addition of a large, bulky anionic backbone is explored by adding a polystyrene‐derived anionic PIL to an MC gel. Some observed differences are that the addition of [Pyr14][TFSI] produced a different gel behavior with phase separation occurring at r.t., especially for higher LiTFSI content (≥1.0 M), while P[DADMA][TFSI] at the same concentration of the [Pyr14][TFSI] sample produced a much more visually observable dense gel.

**Figure 3:**
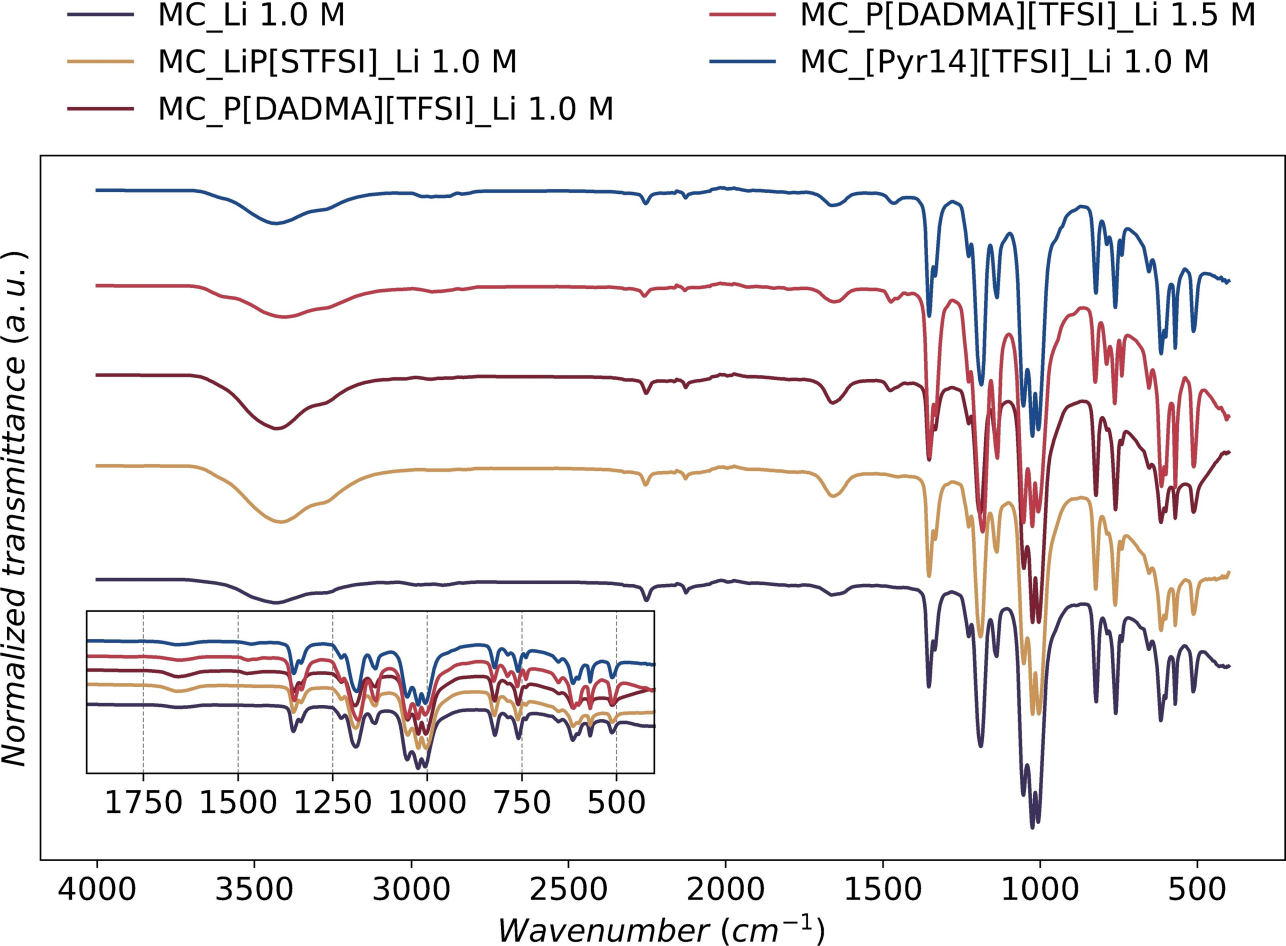
ATR‐FTIR spectra of MC‐based GPEs. Inset showing the expansion of the 400–1750 cm^−1^ region. Full‐size figure is presented in the supporting info.

ATR‐FTIR measurements were done on the MC GPEs to study the effects of the electrolyte composition on cellulose gel structure and Li coordination (Figure 3 and supporting information, Figure S3 and S4). The regular vibration for the stretching of cellulose′s OH occurs at 3400 cm^−1^ and 1100 cm^−1^ for the CH groups.^[43]^ Adding an IL or PIL causes a change in the OH normal vibrations, most probably due to the establishment of H‐bond interactions. Analyzing the spectra in Figure 3, the most significant vibration shift from the MC_Li 1.0 M sample occurs for MC_LiP[STFSI]_Li 1.0 M and MC_P[DADMA][TFSI]_Li 1.5 M, from 3434 cm^−1^ to 3412 and 3400 cm^−1^, respectively. The other samples remain primarily unchanged. The largest shifts observed are likely due to establishing H‐bonds between the MC‐OH groups and SO_2_ groups from [TFSI]^−^.^[44]^ Therefore, the use of an anionic PIL and the increment of the concentration of the LiTFSI promote further H‐bonds within the gel. Although there is a possible difference in hygroscopic behavior between samples, the observed vibration of the OH region reflects the influence of the added PIL or IL.

The vibration bands of the Li salt anion ([TFSI]^−^) were examined to see how different GPE compositions influence the respective shifting. [TFSI]^−^ normal vibration modes are expected to fall between 1300 and 1350 cm^−1^ for the antisymmetric stretching mode of the νaSO_2_ groups and between 1120 and 1160 cm^−1^ for the symmetric vibration, νsSO_2_. The normal vibration modes for the CF_3_ group are expected to occur between 1160 and 1270 cm^−1^, with an intense band and shoulder due to CF_3_ asymmetric vibration, νaCF_3_.^[45]^

In the MC‐based GPEs prepared in this study, shifts in the [TFSI]^−^ vibration bands were observed in the different compositions. Compared to the MC_Li 1.0 M GPE, the vibration of CF_3_ group shifts from 1188 to 1182 cm^−1^ in MC_P[DADMA][TFSI]_Li 1.5 M GPE. The addition of a higher concentration of LiTFSI to a P[DADMA][TFSI] sample shifts the band to slightly lower wavenumber. A similar observation was also reported in literature, with the lower wavenumber band showing higher intensity as well.^[46]^ The band at 1354 cm^−1^, which is assigned to the vibration of SO_2_ groups in MC_Li 1.0 M, suffers a slight shift in P[DADMA][TFSI] _Li 1.5 M GPE, vibrating at 1350 cm^−1^. This is associated with a small weakening of that bond. Furthermore, the peak centered at 740 cm^−1^, which is assigned to an S‐N‐S stretching vibration of free [TFSI]^−^, is a suitable probe for the study of ion pairing.[^47^, ^48^] Vibrations associated with coordinated [TFSI]^−^ were observed for all samples at 760 cm^−1^. The increment of LiTFSI concentration causes another vibration to be observed at 740 cm^−1^. This finding aligns with a previous report indicating that the increase in Li salt led to an increase in the presence of uncoordinated [TFSI]^−^, which agrees with the displacement of the SO_2_ vibration band.

^7^Li‐^1^H and ^19^F‐^1^H Heteronuclear Overhauser effect spectroscopy (HOESY) was used to examine the interactions between Li^+^ and [TFSI]^−^ with [Pyr_14_]^+^ in the [Pyr_14_][TFSI] containing GPE (see supporting information). HOESY interactions indicate the proximity between the observed nuclei. The intensity of these correlations depends on the number of nuclei involved in the interaction and is proportional to their closeness. Stronger interactions suggest that the nuclei are nearer to one another. From these experiments, it is possible to observe that Li^+^ interacts mainly with certain proton nuclei in the organic cation, while the [TFSI]^−^ interacts with all the proton nuclei. This suggests that the larger anion, [TFSI]^−^, tends to have more HOESY contacts and shows a preference for solvation in the coordination shell, revealing the anticipated size differences between Li^+^ and [TFSI]^−^. These observations validate that LiX salts enable short‐range contacts with the organic cation in ^7^Li‐^1^H interactions, while exhibiting long‐range interactions in ^19^F‐^1^H interactions.^[49]^ In the MC_[Pyr_14_][TFSI] GPEs here analyzed through this technique, the evidence points to lithium ions being coordinated by [TFSI]^−^ and also [Pyr_14_]^+^.

Pulsed‐field gradient nuclear magnetic resonance (PFG‐NMR) was utilized to investigate the electrolyte components. The study measured the MC diffusion to assess the impact of IL, PIL, and LiTFSI additions on polymer dynamics and to assess the mobility of charged species in the GPE.

The obtained GPEs were studied by ^7^Li and ^19^F PFG‐NMR diffusion (see supporting information), and the obtained diffusion coefficients were used to calculate the Li^+^ transference number, according to $t^{+}=\frac{D^{+}}{\sum D}$ .

Commercial carbonate‐based electrolytes were also analyzed in comparison to the GPEs.

**Figure 4:**
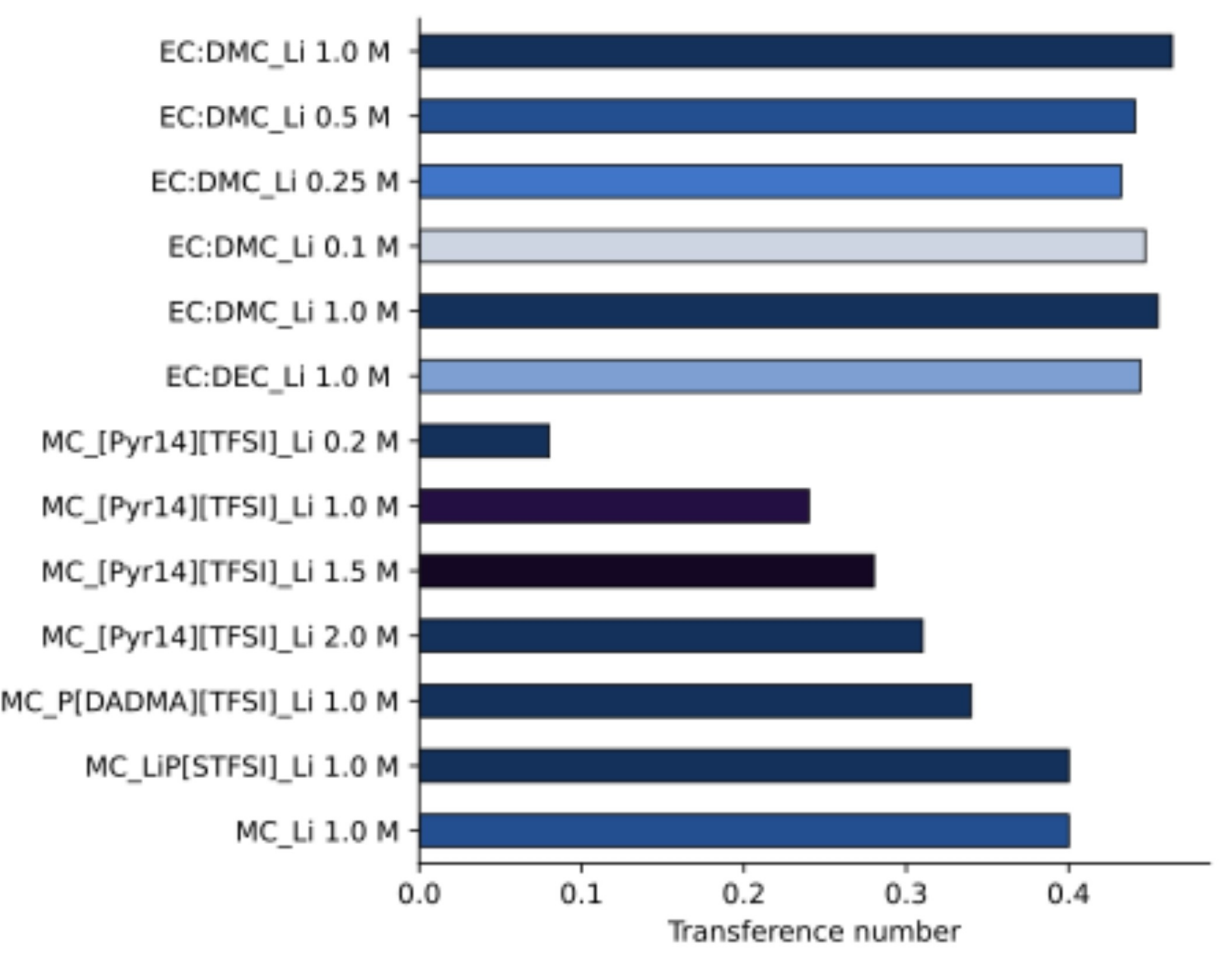
PFG‐NMR determined tLi+ for liquid and gel electrolytes.

The t_Li_^+^ are shown in Figure 4. Compared to liquid electrolytes, gel systems usually have lower ionic conductivity and lower transference numbers. Increasing the amount of Li salt usually increases the transference numbers as seen in the nearly threefold increase in MC_[Pyr14][TFSI] GPEs with 0.2 M and 1.0 M LiTFSI. The current research demonstrates the possibility of achieving similar t_Li_^+^ values for some GPEs compared to liquid electrolytes. When using ILs, as [Pyr14][TFSI], the high mobility of both the IL′s anion and the cation promotes the increase in t_Li_^+^ with increasing LITFSI content from 0.2 to 2.0 M, but never reaching similar values to the liquid electrolytes. In MC_[Pyr14][TFSI]_2.0 M LiTFSI, the higher lithium salt concentration results in a more viscous‐like sample instead of a gel, nevertheless, probably due to lithium being so effectively coordinated, as evidenced by the HOESY experiments, t_Li_^+^ remains limited.

Overall, this calculation shows that higher levels of t_Li_+ can be achieved by using less charged species, such as in MC_Li 1.0 M GPE, or some GPEs like MC_LiP[STFSI] and MC_P[DADMA][TFSI]. In these samples, lithium can move faster, probably because it establishes fewer or weaker interactions and t_Li_^+^ becomes higher. A similar behavior has been described in cellulose acetate‐derived electrolytes that are capable of promoting Li‐salt dissociation and favoring Li‐ion transfer.^[50]^

The t_Li_+ achieved with MC_LiP[STFSI] and MC_P[DADMA][TFSI] GPEs of 0.40 are comparable with the GPEs described for lithium‐ion batteries using unmodified MC and the respective composites that span between 0.25 and 0.68.^[51]^

**Figure 5:**
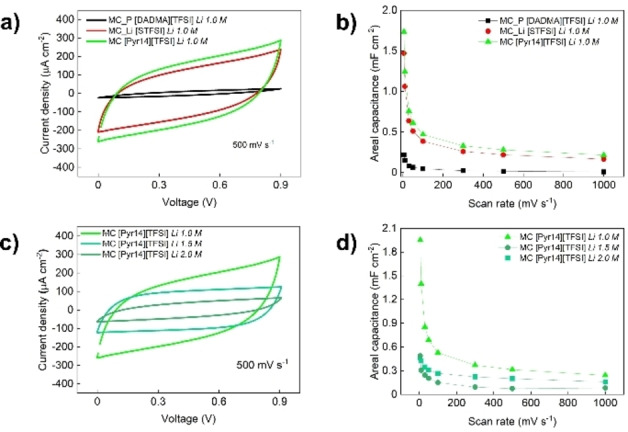
Cyclic voltammograms (a) and respective areal capacitance as function of the scan rate (b) for the different electrolytes under testing with a 1.0 M concentration of LiTFSI; Cyclic voltammograms (c) and respective areal capacitance as function of the scan rate (d) for the MC[Pyr14][TFSI] electrolytes with different amounts of LiTFSI.

As proof of concept, the GPEs were cast in a microsupercapacitor (MSC), which was created using laser‐induced graphene (LIG)[^52^, ^53^] on kapton to produce an interdigitated MSC, (see supporting information Figure S5). The gel electrolytes were applied to the devices and allowed to diffuse before conducting electrochemical testing using cyclic voltammetry. The results of these experiments are presented in Figure 5.

For all the electrolytes, the typical “squared‐shape” cyclic voltammetry was observed, which is expected for carbon‐based MSC. However, it is clear that for MC/PDADMA the capacitive performance is negligible at 500 mVs^−1^ (Figure 5a). Even at a lower scan rate, the areal capacitance for this electrolyte is relatively lower than for the other systems under study (Figure 5b). Nevertheless, the obtained capacitance is similar to what can be expected for a planar graphene MSC. This result suggests that MC/PDADMA does not penetrate the LIG structure, resulting in a pure surface effect. For MC/P[STFSI] and MC/[Pyr14][TFSI], the higher capacitance values are characteristic of porous structures, such as LIG, where the higher surface area results in better electrochemical performances. The cyclic voltammetry results may also be dependent on the ionic conductivity within the electrolyte, which affects the overall capacitance. Regarding lithium concentration in the electrolyte (Figure 5c and Figure 5d) the MSC exhibits a similar capacitive response. For LiTFSI 1.0 M, the obtained CV is quite like the results for conventionally used PVA based electrolytes (Figure S6). However, as the lithium concentration increases, the device performance gets worse. Above this LiTFSI concentration, MC_[Pyr14][TFSI] GPEs become more viscous‐like which leads to less structured samples where MC besides all the ionic species can be partially in the more mobile phase of the gel, leading to lithium ions that although mobile, are much more engaged in interactions, decreasing the performance of the respective devices. This study provides a comparative analysis of various electrolytes and their influence on LIG‐based MSC performance, revealing promising insights into electrolyte optimization for EES applications. While further electrochemical testing and stability assessments are necessary to fully optimize and validate electrolyte functionality, the results presented here underscore the potential of the prepared electrolytes to advance MSC design and enhance energy storage capabilities.

## Conclusions

MC was successfully used as the backbone for GPE production with the use of an IL, PILs, and LiTFSI as a dopant. The study focused on the differences in Li^+^ coordination with [TFSI]^–^ by utilizing ATR‐FTIR, which revealed the impact of the IL and different PILs on free [TFSI]^–^, notably, in sample MC_P[DADMA][TFSI]_Li at a LiTFSI concentration of 1.5 M. The concentration of LiTFSI was a critical factor influencing the obtained results. The PFG‐NMR diffusion techniques were employed to calculate the Li^+^ transference number, providing a comparison between the prepared GPEs and commercial electrolytes. Despite variations in viscosity, the combination of slow diffusing PILs and fast diffusing Li salt achieved comparable Li^+^ transference numbers to those of liquid electrolytes. Specifically, the use of an anionic PIL, LiP[STFSI], demonstrated the potential to enhance the Li^+^ transference number, indicating that the nature of the polymer can effectively modulate Li^+^ transport within a GPE. MC GPEs exhibit significant potential for application in supercapacitors. The cyclic voltammetry analysis showed a rectangular profile, typical for capacitive behavior, although the maximum current was lower than that of proton‐based systems. These findings suggest that further optimization of the GPE composition could enhance their performance, paving the way for their use in sustainable and efficient energy storage systems. Future work should focus on optimizing the concentration and composition of PILs to further enhance ion mobility and conductivity in GPEs. Additionally, investigating the long‐term stability and performance of these GPEs in practical energy storage devices will be crucial for their successful commercialization.

## Conflict of Interests

The authors declare no conflict of interest.

1

## Supporting information

As a service to our authors and readers, this journal provides supporting information supplied by the authors. Such materials are peer reviewed and may be re‐organized for online delivery, but are not copy‐edited or typeset. Technical support issues arising from supporting information (other than missing files) should be addressed to the authors.

Supporting Information

## Data Availability

The data that support the findings of this study are available from the corresponding author upon reasonable request.
