# Supplementary material for: Poly(Ionic) Liquid‐Enhanced Ion Dynamics in Cellulose‐Derived Gel Polymer Electrolytes
Source: ChemSusChem. 2024 Nov 26;18(6):e202401710. doi: 10.1002/cssc.202401710 (PMC11912104; doi:10.1002/cssc.202401710)
Supplement: Supplementary file 1 — Supporting Information [file CSSC-18-e202401710-s001.pdf]

ChemSusChem

Supporting Information

Poly(Ionic) Liquid-Enhanced Ion Dynamics in Cellulose-Derived Gel Polymer Electrolytes

Tiago G. Paiva,* Maykel Klem, Sara L. Silvestre, João Coelho, Neri Alves, Elvira Fortunato, Eurico J. Cabrita, and Marta C. Corvo*

SUPPORTING INFORMATION

Poly(ionic) Liquid-Enhanced Ion Dynamics in Cellulose-Derived Gel Polymer Electrolytes

Tiago G. Paiva[a,b]*, Maykel Klem[a,c], Sara L. Silvestre[a], João Coelho[a,d], Neri Alves[c], Elvira Fortunato[a], Eurico J. Cabrita[e], Marta C. Corvo[a]*

[a] I3N|Cenimat, Department of Materials Science (DCM)
NOVA School of Science and Technology, NOVA University of Lisbon
Caparica 2829-516, Portugal
[b] Centro de Química Estrutural, Institute of Molecular Sciences and Departamento de Engenharia Química
Instituto Superior Técnico, Universidade de Lisboa
Avenida Rovisco Pais 1049-001 Lisboa, Portugal
[c] School of Technology and Sciences, São Paulo State University (UNESP)
Presidente Prudente, SP 19060-900, Brazil
[d] Instituto de Ciencia de Materiales de Sevilla (Universidad de Sevilla-CSIC
Avda. Americo Vespucio 49, Sevilla 41092, Spain
[e] UCIBIO, Department of Chemistry
NOVA School of Science and Technology, NOVA University of Lisbon
Caparica 2829-516, Portugal

Corresponding authors: tiagogpaiva@tecnico.ulisboa.pt; marta.corvo@fct.unl.pt

Table of Contents

1 Materials and methods **2**

1.1 Materials **2**

1.2 Methods **2**

1.2.1 NMR spectroscopy 2

1.2.2 ATR-FTIR 5

2 Synthesis **6**

2.1 Preparation of P[DADMA][TFSI] **6**

2.2 Preparation of LiP[STFSI] **6**

3 Laser Processing and MSC testing **7**

4 References **8**

1 Materials and methods

1.1 Materials

MC (400 Cp) was purchased from Sigma-Aldrich. MC was dried at 70 °C overnight before use. 4-*tert*-Butylcatechol (TBC) 98 %, triethylamine (99 %), Poly(diallyldimethylam- monium) chloride (PDADMAC) 20 wt. % solution (average Mw 200,000-350,000) were obtained from Sigma-Aldrich. AIBN from Glentham Life Sciences. Trifluoromethanesulfonamide was purchased from TCI Chemicals. DMF and toluene were purchased from Sigma-Aldrich, distilled and stored with molecular sieves(Linde type 3 Å, Sigma-Aldrich). Acetonitrile was purchased from Carlo Erba and used as received. Dimethyl sulfoxide (DMSO), as well as deuterated dimethyl sulfoxide - (DMSO-d_6), were obtained from Eurisotop. [Pyr_{14}][TFSI], LiTFSI, DMC, DEC, EC/DMC 25/75 1 M LiTFSI and EC/DEC 50/50 1 M LiTFSI were purchased from Iolytec and used as received.

1.2 Methods

1.2.1 NMR spectroscopy

A Bruker Avance III 400 operating at magnetic field 9.4 T, corresponding to Larmor frequencies of 400 MHz, 386 MHz and 155 MHz for ^1H, ^{19}F, and ^7Li respectively, was used to obtain the PFG-NMR data. A water-cooled DiffBB probe driven by a GREAT60 amplifier capable of 17 Tm^{-1} gradient strength was used in all measurements.
Each experiment was carried out in 16-32 magnetic field gradient steps, accumulating 14k points in 16-128 transients. Diffusion time (Δ) was a fixed value of 20 ms or varied between 20 to 200 ms in selected experiments. The gradient pulse duration (δ) was 1 ms except where noted, the maximum B value was calibrated to achieve 95 % attenuation while retaining signal above the noise threshold. At the beginning of each experiment, a 1D measurement of t_{null} was done to calibrate the recycle delay, which was set to 3-5 times the T_1. A spoil gradient was used to remove unwanted magnetization.

1.2.1.1 HOESY

HOESY spectra were acquired using the Bruker hoesyph pulse sequence using 100, 300 and 500 ms mixing times. ^7Li-^1H spectra were adquired in the DiffBB probe,

a sweep width of 8012.82 x 4000.00 Hz was sampled in 2048 x 128 points acquired in 16 transients. ^{19}F-^{1}H HOESY spectra were acquired using a BBFO probe, a sweep width of 5597.02 x 2000.00 Hz was sampled in 2048 x 128 points acquired in 16 transients.

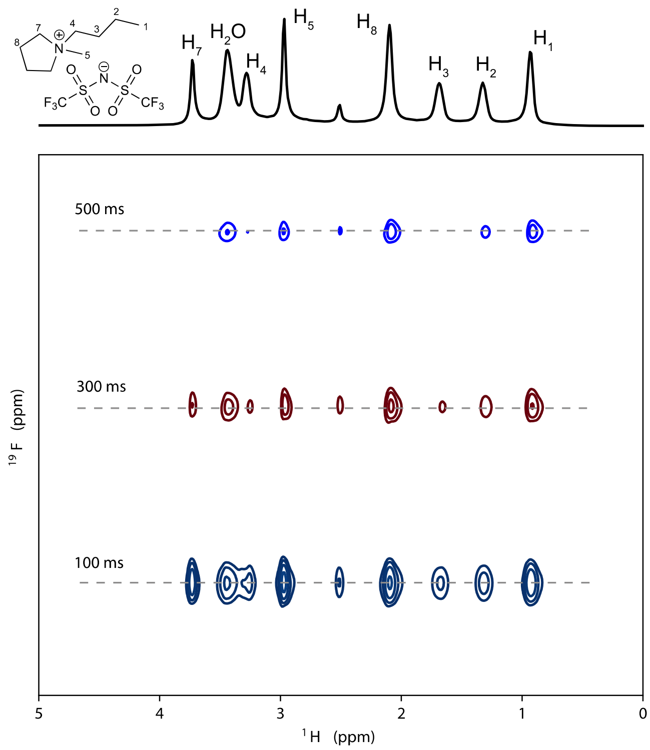

Figure S1. ^{19}F – ^{1}H HOESY spectra of the MC 3 wt. % [Pyr$_{14}$][TFSI] containing GPE at 100, 300 and 500 ms mixing times.

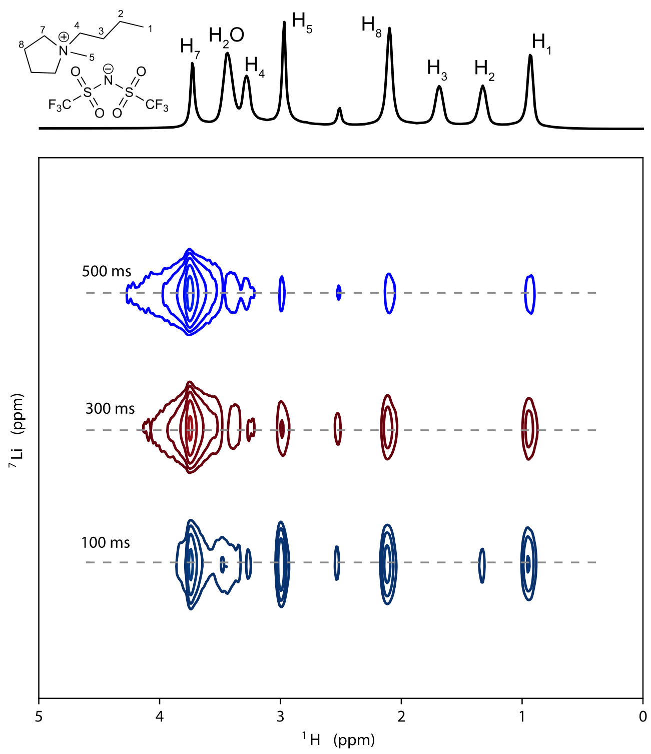

Figure S2 ^{7}*Li* – ^{1}*H* HOESY spectra of the MC 3 wt. % [Pyr14][TFSI] containing GPE at 100, 300 and 500 ms mixing times.

1.2.1.2 PFG-NMR

PFG-NMR diffusivity values for the studied GPEs charged components are presented in Table S1.

Table S1 Charged species PFG-NMR diffusivities and calculated t_{Li}^{+} *for the studied GPEs*

D ($m^2 s^{-1}$)	IL/PIL cation/ STFSI anion	$^{7}Li^{+}$	(^{19}F) $TFSI^{-}$	t_{Li}^{+}
EC:DMC_Li 1.0 M		1.98E-10	2.29E-10	0.46
EC:DMC_Li 0.5 M		2.69E-10	3.41E-10	0.44
EC:DMC_Li 0.25 M		3.61E-10	4.74E-10	0.43
EC:DMC_Li 0.1 M		4.40E-10	5.44E-10	0.45
EC:DMC_Li 1.0 M		2.73E-10	3.27E-10	0.46
EC:DEC_Li 1.0 M		1.71E-10	2.14E-10	0.44
MC_[Pyr14][TFSI]_DMSO_Li 0.2 M	2.28E-10	1.69E-10	2.64E-10	0.08
MC_[Pyr14][TFSI]_DMSO_Li 1.0 M	1.75E-10	1.28E-10	1.83E-10	0.24
MC_[Pyr14][TFSI]_DMSO_Li 1.5 M	1.51E-10	7.99E-11	1.07E-10	0.28
MC_[Pyr14][TFSI]_DMSO_Li 2.0 M	1.13E-10	1.08E-10	1.45E-10	0.31
MC_P[DADMA][TFSI]_DMSO_Li 1.0 M	1.87E-12	1.27E-10	1.52E-10	0.34
MC_LiP[STFSI]_DMSO_Li 1.0 M	6.00E-12	1.42E-10	2.09E-10	0.40
MC_DMSO_Li 1.0 M		1.51E-10	2.26E-10	0.40

1.2.2 ATR-FTIR

ATR-FTIR data was collected in a PerkinElmer Spectrum Two FTIR spectrometer equipped with a Universal Attenuated Total Reflectance (μATR) accessory. Each spectrum was collected from 400 to 4000 cm^{-1} in 8 scans.

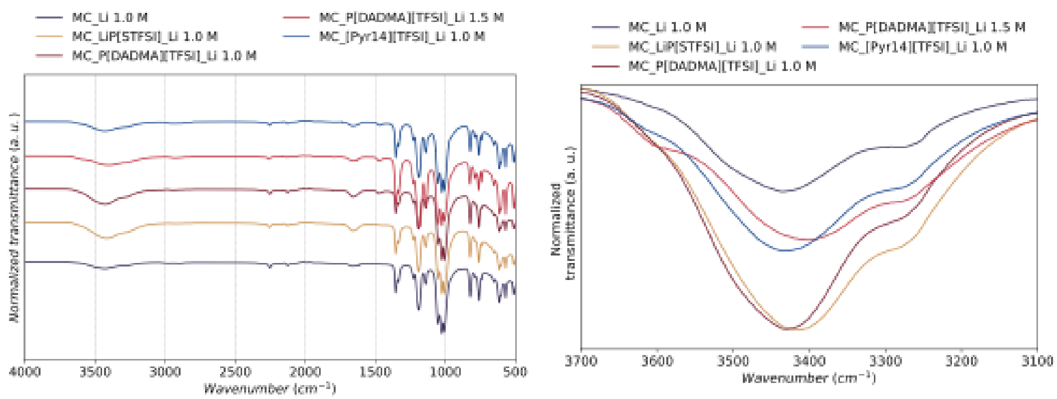

Figure S3. ATR -FTIR spectra of MC-based GPEs shown opposite to an expansion for the 3700 – 3100 cm^{-1} region.

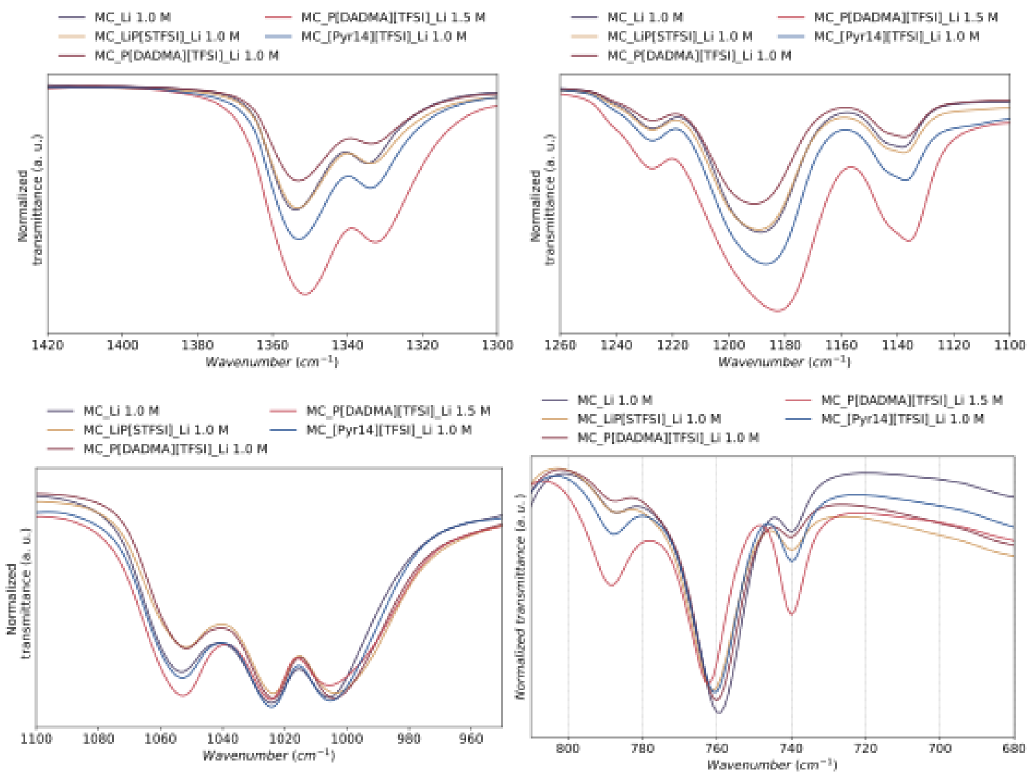

Figure S4. Expansions of the 1420 – 1300 cm^{-1}, 1260 – 1100 cm^{-1}, 1100 – 950 cm^{-1} and 810 – 680 cm^{-1} regions of the ATR-FTIR spectra of MC-based GPEs.

2 Synthesis

2.1 Preparation of P[DADMA][TFSI]

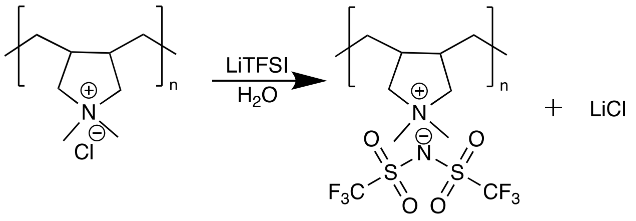

P[DADMA][TFSI] was prepared from ion metathesis reaction from P[DADMA]Cl. 20 wt. % in water (0.654 g, 3.95 mmol) were added dropwise to a 1 mol L^{-1} solution of LiTFSI in water. The product precipitated from water was then filtered and washed with cold water, P[DADMA][TFSI] was achieved in a 65 % yield.

^{1}H-NMR (400 MHz, DMSO-d_6) δ 3.70, 3.19, 3.16, 3.08. ^{19}F-NMR (376 MHz,(DMSO-d_6)) δ -78.65. ^{13}C-NMR (100 MHz, (DMSO-d_6)) δ 52.15, 54.08, 54.74.

2.2 Preparation of LiP[STFSI]

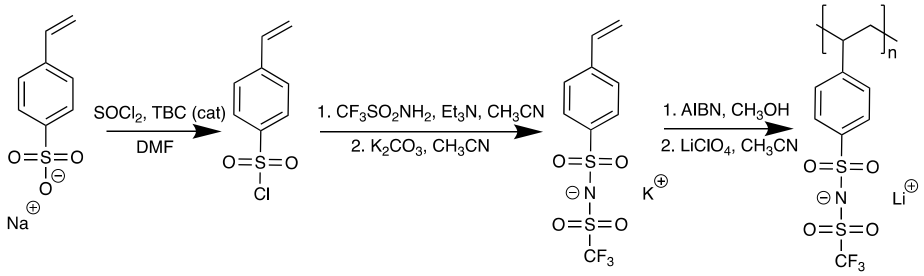

The following procedure was adapted from two literature sources [1,2]. Sodium 4-vinylbenzenesulfonate (0.8 g, 4 mmol) and 4-tert-butylcatechol (20 mg) were added drop- wise in 6 mL of DMF under N_2 to 10 mL of thionyl chloride under stirring in an ice-bath The resulting suspension was allowed to reach r.t. and kept under stirring for an additional 3 h until a clear solution was obtained. The reaction was quenched by diluting with cold H_2O (15 mL) and the flask was placed overnight at 4 °C. Afterwards, the product was extracted with toluene, (2×10ml) and then washed with H_2O (2×10mL). The organic phase was collected and concentrated at 50 °C to produce a yellow liquid, 4-vinylbenzenesulfonyl chloride. The compound was subsequently dissolved in (10 mL) acetonitrile and added (1.3 mL) of TEA and

trifluoromethanesulfonamide (0.6 g). The reaction was stirred for 48 h at r.t. The solvent was evaporated to remove volatiles, and the obtained solid residue was extracted using dichloromethane (DCM) (10 mL) and washed with H_2O (2×3 mL). The DCM layer was collected and concentrated in a rotary evaporator to afford a viscous liquid, which was neutralized by excess Na_2CO_3 in anhydrous methanol (10 mL). Methanol was filtered and evaporated to afford Na[STFSI] as an off-white solid. ((4-(*sec*- Butyl)phenyl)sulfonyl)((trifluoromethyl)sulfonyl)amide, LiP[STFSI], was prepared by free radical polymerization in methanol followed by ion exchange reaction with $LiClO_4$ in acetonitrile.

^{1}H-NMR (400 MHz, (Methanol-d_4) δ 7.75, 6.66, 1.56. ^{19}F-NMR (376 MHz, Methanol-d_4) δ -78.65. ^{7}Li-NMR (155 MHz, Methanol-d_4) δ 0.38.

3 Laser Processing and MSC testing

The MSC were assembled by printing interdigital electrodes on PI with laser power of 8.5 W at a scanning speed of 0.14 m s^{-1}. MSCs on PI were characterized in a BioLogic SP-50 potentiostat (BioLogic Sciences Instruments) by means of cyclic voltammetry (5 to 1 V s^{-1}). Prior to electrolyte casting, the MSCs were cured under UV light (NOVASCAN-PSD Pro Series Digital UV Ozone System) for 15 min at room temperature to reduce the hydrophobicity of the LIG. The electrolyte was then drop-cast onto the devices over an area of ~1 cm^2. The assembled MSCs were allowed to dry overnight at room temperature. In Figure S5 it is shown a representative image of the produced devices.

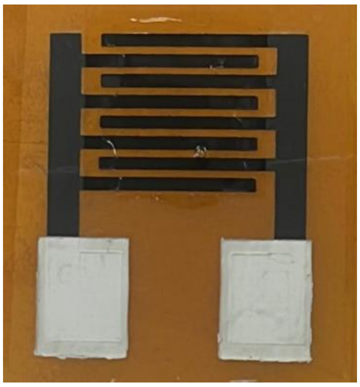

Figure S5. LIG - MSC on kapton.

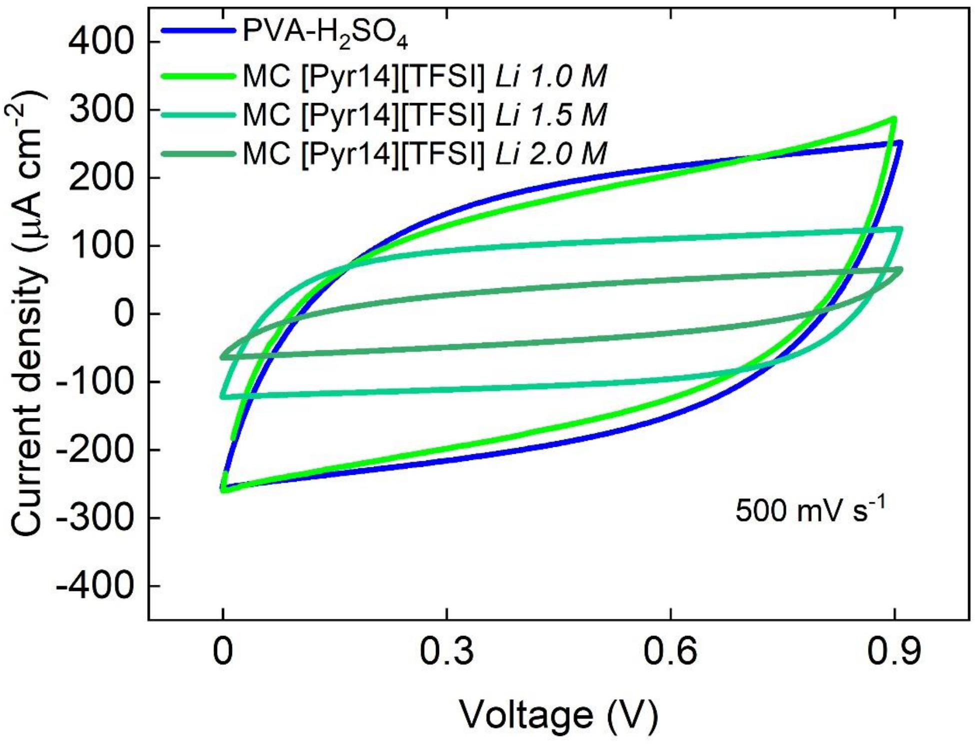

Figure S6. LIG - MSC cyclic voltammetry comparing the voltammetry curves for devices with PVA/H2SO4 as electrolyte with electrolytes exhibiting different amounts of lithium.

4 References

[1] Q. Ma, H. Zhang, C. Zhou, L. Zheng, P. Cheng, J. Nie, W. Feng, Y.-S. Hu, H. Li, X. Huang, L. Chen, M. Armand, Z. Zhou, *Angew. Chem. Int. Ed.* **2016**, *55*, 2521–2525.

[2] J. Li, H. Zhu, X. Wang, M. Armand, D. R. MacFarlane, M. Forsyth, *Electrochimica Acta* **2015**, *175*, 232–239.
